# Financial Development and Environmental Degradation: Promoting Low-Carbon Competitiveness in E7 Economies’ Industries

**DOI:** 10.3390/ijerph192316336

**Published:** 2022-12-06

**Authors:** Guohua Liu, Mohammed Arshad Khan, Ahsanuddin Haider, Moin Uddin

**Affiliations:** 1 Accounting Faculty, Hebei Vocational University of Technology and Engineering, Xingtai 054000, China; 2 Department of Accountancy, College of Administrative and Financial Sciences, Saudi Electronic University, Riyadh 11673, Saudi Arabia; 3 Department of Finance, College of Administration and Financial Science, Saudi Electronic University, Riyadh 11673, Saudi Arabia

**Keywords:** low carbon, ecological footprint, ecological quality, fostering environmental sustainability

## Abstract

Emerging countries are approaching economic prosperity. However, the development process has enhanced their ecological footprints, thus promoting low-carbon competitiveness among E7 countries’ industries. Therefore, it is essential to identify the factors that affect a country’s ecological footprint (EF) in order to safeguard the environment. This study explored the effect of financial development, human capital, and institutional quality on the EF of emerging countries. Furthermore, we explored the effect of financial development on the EF of emerging countries through the human capital channel. In addition, we investigated the role of institutional quality in the financial development–EF nexus. Using panel data from 1990 to 2018, we employed the cross-sectional autoregressive distributed lag (CS-ARDL) technique to conduct a short-term and long-term empirical analysis. The empirical outcomes revealed that financial development degrades ecological quality by raising the EF. The findings further demonstrated that human capital and institutional quality reduce the EF. Moreover, financial development fosters environmental sustainability through the channel of human capital. Additionally, institutional quality reduces the negative ecological impacts of financial development. The causality analysis suggested that any policy related to financial development, human capital, and institutional quality will affect the EF. However, the inverse conclusion was not sustained. Based on these findings, emerging economies should increase their environmental sustainability by promoting human capital and effectively using financial resources.

## 1. Introduction

The greenhouse effect has recently been linked to several natural environmental issues, including sea level rises, glacial retreats, and global warming. The global average surface temperature has increased by 0.85 °C, representing a 4.4% to 5.1% change, over the last century, having increased by 0.19 from 1902 to 2011 according to an Intergovernmental Panel on Climate Change study [1]. High levels of CO_2_ emissions are the primary cause of the greenhouse effect [2]. The E7 countries have an undoubted level of responsibility for the worsening of the environment because they overtook the G7 countries as the largest emitters of CO_2_ in 2008. As a result, the E7 countries have committed to increasing their energy efficiency, lowering their use of coal, and reducing their CO_2_ emissions. The E7 governments pledged, during the Copenhagen Global Warming Conference in 2008, that the CO_2_ emissions per unit of E7 gross domestic product would fall by 40% to 45% by 2019 [1]. In addition, the G7 governments promised that their countries would aim to reach peak CO_2_ emissions by the year 2040. The E7 countries face a difficult development situation as a result of their growing urbanization. Between 1970 and 2014, the E7 countries’ share of the urban population increased from 19.39% to 56.10% [3], which represented a rise of roughly 1.05% each year, and this figure approached 60% by 2019. The E7 countries are still in the second stage of urbanization according to the theory of the development of urbanization presented in [4]. E7 urbanization has a long way to go as urbanization in wealthy nations has only recently reached its third stage. Additionally, the authors of [5] argued that hundreds of millions of rural dwellers will move to metropolitan regions in the future, changing the population demographics and the associated energy use and CO_2_ emissions of manufacturers [6]. First, as individuals’ habits change, there will be an increase in CO_2_ emissions from clothing, food, shelter, and transportation. Second, the shift from agricultural to industrial manufacturing will inevitably result in increased infrastructural development and industrial manufacturing, both of which will increase CO_2_ emissions.

The industrialized E7 nations are steadfastly sustaining their economies’ expansion through the expansion of their trade and production industries [7]. The E7 countries are regarded as the second-largest polluters in the world after the G7 countries, and their strict focus on economic activities has degraded the environment. Consequently, the incidence of environmental problems is rapidly increasing [8]. However, programs and measures have been put in place to effectively support both environmental sustainability and financial development [9]. Nevertheless, the results reported for these programs are not reliable, and research that does not take the composite risk index into account offers conflicting conclusions about environmental measures [10]. Investigating these issues and providing environmental, economic, and policy-oriented recommendations that pertain to both the economy and the environment are therefore crucial tasks [11]. Both environmental and financial indices are influenced by numerous factors. The composite risk index is one of these factors, and it is reported to have a significant environmental impact [12]. Countries are more likely to switch to environmentally friendly resource usage if the composite risk index is lower, boosting environmental sustainability [13]. Strict environmental rules encourage a new competitive landscape, or the adoption of green innovation, in industrialized countries [14]. The manufacture of environmentally friendly products and services is referred to as “green innovation”, and, even at the business level, the number of green consumers is increasing rapidly [15]. In contrast, businesses and end-users ignored environmental sustainability during the pre-green technology era, which significantly contributed to environmental degradation [16]. To achieve carbon neutrality, manufacturing companies—especially those in developed economies—need to adopt green innovations that reduce the negative effects of the production process [17].

One of the most important strategies for advancing green development is financial support. Through “capabilities of credit creation” and “resource-allocation capabilities”, financial organizations can support economic growth, according to the “financial development theory” put forth by Schumpeter and the “financial structure theory” proposed by Goldsmith in the middle of the 20th century [18]. The relationship between financial development and green financial activities further demonstrates the dual function of the financial sector [19]. On the one hand, the “scale effect”, “structure effect”, and “technology effect” of finance can broaden the economic scale [20]. By modernizing machinery and manufacturing processes and using more environmentally friendly production techniques, financial development can enhance the financial architecture and reduce environmental pollution [21]. On the other hand, the expansion of funding sources for businesses with high energy use, emissions, and pollution due to financial improvements has decreased the effectiveness of green financial development overall [22].

From the standpoint of policymakers, it is essential to achieve stable financial investment and import efficient technology for green energy projects, but it is equally necessary not to lose sight of environmental degradation concerns. For the success of SDGs 7 and 13, this is an essential component. The socioeconomic upheaval of a country has an effect on international relations. In this regard, there may be inconsistencies when it comes to spending on environmentally friendly initiatives and tapping into the inventive energy of emerging nations. Green and renewable energy projects, which improve the ecological environment by reducing carbon emission (CO_2_) levels, may demonstrate this effect. As a result of these changes, business sectors also have an impact on environmental degradation. The involvement of policymakers is vital in reducing the improbability of environmental issues due to shocks. The primary goal of this empirical analysis is to suggest these policy solutions for E7 nations, and the research objective is framed below.

Maintaining economic development while simultaneously finding energy solutions (such as increasing the share of renewable energy projects) to fulfil demand and enhance ecological integrity is crucial. Financial sector development (FD) is vital for the economic progress of E7 nations because it allows low-interest loans to be issued to businesses and households. The subsequent increase in energy use and thus pollution are direct results of this phenomenon. Due to their advanced state of conventional industrialization and heavy use of fossil resources, E7 nations are experiencing climate change effects; the only solution is to discover a new, sustainable power source (green or renewable energy). Several policies need to be implemented for this transformation, despite its apparent simplicity. One of the greatest problems is the degree of risk relating to invested financial capital for green or sustainable energy development, as the consensual aspirations of governments can often be compromised by factors such as constant innovation in technical capabilities, the sharing of money, poor financial mechanisms, the first-time financing of such developments (new entry point), and fewer government subsidies. To accomplish the SDGs, it may be necessary to take on financial risk when investing in green or renewable projects, a point that is endorsed by the Green Development Guidance for E7 Projects Baseline Study Report 2020.

Monetary progress has been cited as both the root of the problem and the key to resolving the environmental concerns brought about by the emission of greenhouse gasses. One way in which the environment suffers is due to the coal consumption that is the result of global financial expansion, which in turn encourages credit expansion, investments, and wealth creation. However, the adverse effects of greenhouse gases are mitigated by financial development, which makes it possible to invest in energy-saving machinery and technology.

Improved corporate management is one way in which increased financial growth benefits the environment. Businesses with good corporate governance practices are more inclined to use efficient environmental management policies. Economic performance, according to a key study, may enhance environmental circumstances because it encourages the entrance of multinational corporations’ expenditures, which are often linked to considerable research and development operations. Scholars have shown a great deal of empirical interest in the factors that determine ecological integrity in SSA in recent times. Examples of research that show that sustainable energy use improves ecological integrity include the finding that economic expansion, urbanization, and the production of quasi-energy all degrade the quality of the environment. Despite the fact that FDI, trade balance, population growth, and economic growth all degrade the quality of the environment, the research also shows that democratization and the use of sustainable sources improve it. Although several studies have been conducted to predict the quality of the environment, the relationship between economic growth and environmental protection in SSA remains poorly understood. Based on the few known research works, it seems that the issue of whether or not economic growth directly affects ecological integrity in SSA is far from established.

It is crucial to record how the studied policy measures have changed the appropriate policy metrics while incorporating the reform agenda. Since this aspect is relevant to the study’s goals, an appropriate methodology was used to investigate it. Furthermore, uncertainty in predicted outcomes might arise from E7 nations’ social and economic cooperation and connection. Due to the need to tackle this issue, this research used a strategy from the second generation. In addition, the review of the literature, conceptual foundation, and research commentary may all be found in Section 2 and Section 3, respectively, of this empirical investigation. In addition, the data sources and macroeconomic approaches are discussed in Section 4, while the results, debates, conclusions, policy suggestions, and limitations are presented in Section 5.

## 2. Literature Review

In the realm of energy and environmental research, the connection between CO_2_ emissions and energy-saving technologies has recently gained attention. Although it is widely accepted that energy-saving innovation may aid in reducing energy use, it is also possible for energy-saving innovation to increase energy use and CO_2_ emissions. As discussed in [23], the energy rebound effect describes this process. The following description details the causes of the rebound effect. Residents’ financial strains will be lessened, and more energy may be utilized, since efficient energy technology makes it possible for customers to obtain the same quantity of services at a cheaper price. After using energy-saving lamps for domestic illumination, inhabitants may cease the practice of randomly turning them off for [24] comfort and aesthetics, which would then cause a rise in energy consumption. This is similar to the way in which homeowners may wish for greater cooling and heating effects as energy-saving air conditioning technology advances, thus increasing power use. With the advancement of automotive production innovation, the amount of fuel used for every mile is decreasing [25]. Residents may travel more often as a result of the development of energy-saving technologies, which would cause a rebound effect [26]. Technologically upgrading gas equipment will increase inhabitants’ effectiveness in using gas, which might potentially result in an over-reliance on gas in terms of usage.

According to a conceptual model developed by [27] and based on the theories of neoclassical finance from the micro viewpoint, energy effectiveness will increase as technology advances, but energy consumption may not necessarily decrease. From a socioeconomic standpoint [28], it aids innovation advancement. As a result, although the advancement of energy-saving technologies might increase, it might not lead to a reduction in energy use and CO_2_ emissions [29]. This demonstrates how technologies may raise energy consumption and boost CO_2_ emissions.

There is a wealth of literature on “carbon neutrality,” one of today’s most contentious problems, as well as its influences on environmental strategy, green innovation, and composite risk indices for many nations and areas around the globe [30]. Carbon dioxide (CO_2_) and other greenhouse gas (GHG) emissions are well known to be major global problems. The focus of academics is largely on carbon dioxide emissions. To achieve carbon-neutral or carbon-free ecosystems, almost all nations have agreed to limit their carbon dioxide emissions [31]. The authors of this study made the case that, in order to achieve carbon neutrality while addressing energy and climate concerns, it is necessary to maximize the potential and complementarity of all available options, including technological advancements, carbon sinks, circular economy initiatives, and changes in mobility patterns.

Ref. [32] studied E7 towns that made climate-related announcements to determine whether the carbon neutrality goal could be accomplished via climate mitigation objectives. The findings, which made use of correlations and statistical measurements, claimed that larger cities are more ambitious about global warming than smaller ones in terms of reaching carbon neutrality [33]. Additionally, it was noted that European towns lacked the necessary resources to adhere to the Paris Agreement, which aims for net-zero emissions by the middle of the twenty-first century. The authors of [34] used statistical analysis to consider E7 countries’ ability to accomplish the carbon neutrality objective via a carbon price and examine environmental policies. The analysis found that the province’s carbon emissions were decreased by between 5% and 15% points as a result of the rise in carbon tax from USD 10 to USD 40.

The failure of the research cited above to take into account how financial growth affects the environment is another significant flaw in such studies. Some studies have only recently taken into account how financial development affects ecological achievement. According to some researchers, financial development could help businesses to achieve greater economies of scale [35], improve manufacturing innovations or manufacturing techniques, promote investments in environmental projects [36], encourage more environmentally friendly behaviors [37], or eliminate backward manufacturing due to an inefficient allocation system. Additionally, according to [38], financial intermediaries may speed up technological advancement, which would reduce pollutant emissions. According to certain research, financial prosperity in E7 countries results in a rise in patents and a favorable impact on technical technology [39]. Some academics, on the other hand, emphasize the detrimental impacts of financial development. According to some investigators, financial development may have encouraged expenditures in industrial projects, led to new entrants in some heavy industries, increased energy usage, and increased waste and pollution [40].

Recent research has sought to examine the total impact of financial development, economic growth, and environmental quality. A non-linear link between money and green development has been shown by some studies. One study discovered that the link between financial development and Green Total Factor Productivity (G-TFP) and marginal efficiency is non-linear and has a dual threshold impact, using E7 province panel data and a threshold regression approach. Additionally, they discovered a favorable “U”-shaped association between G-TFP and environmental administration. According to [41], there is a favorable correlation between the provincial G-TFP and regional financial development in E7 countries, as measured by the spatial Dubin model. The findings of similar non-linear research are also represented by [42]. There are still some particular indications when we focus on the E7 nations. According to a study by [43], foreign direct expenditure may enhance environmental quality, whereas financial growth in E7 nations might worsen it due to CO_2_ emissions. Additionally, the authors discovered that trade liberalization improves environmental quality; energy consumption and urbanization hinder environmental improvement; and economic growth and CO_2_ emissions exhibit an inverted U-shaped curve [44]. In contrast, authors analyzed data from E7 countries to discover that, while financial development and the use of renewable energy have a positive impact on the environment, high-tech industries and financial development have worsened the environmental quality of the E7 countries.

## 3. Methodology

### 3.1. Data Collection

**Figure 1:**
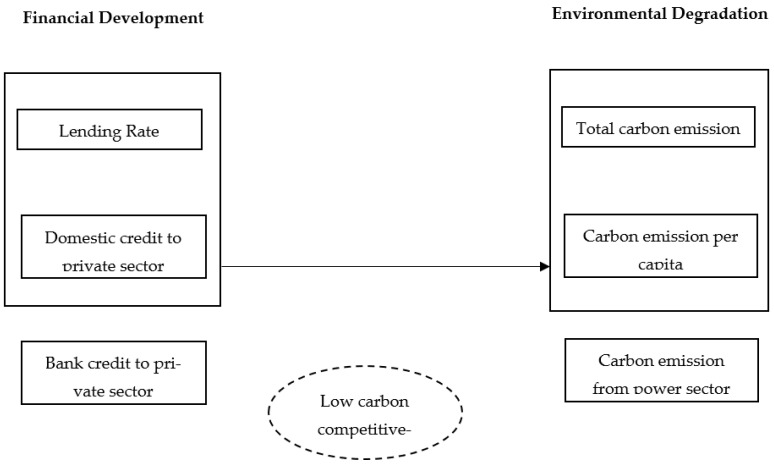
Research framework of the study.

The study investigates the relationship between financial development and environmental degradation in promoting a low-carbon transition for the period from 1990 to 2018. As discussed, the study employed the generalized method of moments (GMM), using its one-step and two-step approach and the seemingly unrelated regression (SUR) test. Variables of the study are described in Figure 1. The study data were acquired from the World Bank database. 

### 3.2. Empirical Models

The study applied the system generalized method of moments (GMM) to test the study framework. This technique has some advantages in terms of estimation, validation, and robustness over other techniques of GMM and is comparatively better than others. However, the econometric form of this is presented in the following equations:

*ED_it_ = a*_0_*+ a*_1_*TCE_it_*_1_*+ a*_2_*CEPC_it_ + a*_3_*CEPPS_it_ + a*_4_*LR_it_ + a*_5_*DC_it_ + a*_6_*BC_it_ ε_it_* (1)

*TCE_it_ = a*_0_*+ a*_1_*LR_it_ + a*_2_*DC_it_ + a*_3_*BC_it_ ε_it_* (1a)

*CEPC_it_ = a*_0_*+ a*_1_*LR_it_ + a*_2_*DC_it_ + a*_3_*BC_it_ ε_it_* (1b)

*CEPPS_it_* = *a*_0_ + *a*_4_*LR_it_* + *a*_5_*DC_it_* + *a*_6_*BC_it_*
*ε_it_* (1c)

Considering the abovementioned equation, two-way GMM estimators are applied. The econometric format of this estimator is written as follows:

*FD_it_ − TCE_i,t −_*_1_*= a*_1_
(*CEPC_i,t−_*_1_ − *CEPPS_i,t−_*_2_) + *a*_2_
(*LR_it_ − LR_i,t−_*_1_) + *a*_3_
(*DC_it_ − DC_i,t−_*_1_) + *a*_4_
(*BC_it_ − BC_E,t−_*_1_) + (*ε_it_ − ε_i,t−_*_1_) (2)

The difference GMM moment condition for Equation (2) is set as below:

      *E*[*FD_i,t_*_−*s*_ ∗ *ε_i,t_*] = 0, for *s* ≥ 2005 *t* = 2005, 2006, …, 2018
*E*[*ED_i,t_*_−*s*_ ∗ (*ε_i,t_* − *ε_i,t_*_−1_)] = 0, for *s* ≥ 2005 (3)

*t* = 2005, 2006, …, 2018 (4)

Parameter estimates using difference GMM may be skewed in small samples and have enormous variance asymptotically. However, as shown in [45], this method helps to control the basis of the issues caused by a country-specific effect and endogenous explanatory factors. Therefore, a system GMM is a viable alternative strategy. For Equation (5), the required system difference GMM moment condition is as follows:

*E*[(*ED_it_*_−*s*_ − *ED_i,t_*_−*s*−1_) ∗ *ε_it_* ] = 0  For *s* = 1, (5)

The one-step and the two-step parameter estimation are variations of the GMM technique found in the literature. Since one-step predictors are theoretically less efficient than two-step estimators, most researchers, including [46], prefer to employ two-step approximation. As a result, the two-stage calculators use weighting matrices that are optimal for the task at hand. However, it should be kept in mind that their use in cross-sections of limited size might result in biased estimators’ characteristics [47]. Nevertheless, the abovementioned issue may not be present in our data set since we employed a large cross-section size.

## 4. Results and Discussion

### 4.1. Empirical Results

The random impacts are rejected at the 10% importance level (*p* = 0.1), as seen in Table 1, indicating that the fixed effects model is more appropriate for the estimation. In addition, the SDM is a suitable option due to the indices of CEPC. In addition to this, pollution in one province has a favorable effect on pollution in neighboring provinces.

To determine how factors in neighboring areas impact the SDM, we consult the weights matrix in [48]. Table 1′s TCE coefficients for CEPC are the most negative and statistically important, showing that rising regional prosperity will lead to lower local SO_2_ emissions. Each of the three pollutant’s TCE and CEPPS coefficients are statistically insignificant. This finding demonstrates that the financial [49] growth of adjacent provinces has no appreciable influence on pollution outputs in the region. Additionally, the geographical impacts of the energy usage per person of the adjacent provinces on pollutant emissions are likely negligible, since all three pollutants have mainly no significant [50] and positive factors of LR. The other geographical impacts of surrounding provinces on the three pollutants are likely to be insignificant and small in scale, as indicated by the fact that they are not statistically important.

When considering geographical dependency, the DC estimates for CEPC and BC in Table 2 are comparable to those in Table 2 [51]. When taking geographical dependency into account, however, the EKC estimates for WW diverge significantly from those in Table 2. This indicates that the coefficients for any [52], for both CEPC and BC, have strongly negative signs. This means that both the predicted connection between CEPC and DC per person and the predicted link among BC and DC per person are “inverted N”-shaped curves. There is also no statistically important BC correlation between this and DC per person.

It is important to note that the coefficients in Table 2 do not indicate the quantitative connection of marginal changes in the dependent and independent factors [53]. Table 2 details the documented direct effects. The CEPC and inflexion points are determined using these coefficients [54]. Using the findings as a measure of economic progress, the initial inflection points for DC per person in CEPC and BC are obtained, while the second inflection points are also calculated [55]. For CEPC per capita, tipping thresholds are obtained, and the second is somewhere between 22,300 and 62,654 yuan, using fineness as the economic growth measure. Table 2 shows that when spatial effects are taken into account, the tipping points for CEPC and DC are both noticeably delayed.

One of the most crucial factors in a country’s economic growth is the richness of its natural resources. Rapid financial development is facilitated by a country’s abundance of natural resources, and the world’s current financial superpowers have all benefited from this resource windfall [56]. There are, however, divergent views on whether a nation’s natural resources are a “gift” or a “curse” in terms of its financial growth and environmental sustainability. On the one hand, conventional economic thought suggests that a nation’s stock of natural resources has a positive effect on its GDP [57]. However, the “resource curse” idea has been verified by several studies [58]. There is a wide disparity in the resource wealth of the E7 nations. To obtain a fuller picture of how each country’s economy has developed, it is important to consider the part that natural resources have played in the country’s financial prosperity and environmentally friendly expansion. Oil, coal, and natural gas form the backbone of many economies [59]. The proven reserves of oil, natural gas, and coal account for a share of the world’s total, and this information was used as a reference in the categorization of resource endowment based on the 2019 BP Statistical of World Energy. For the sake of this discussion, “non-resource endowment” refers only to those nations that lack all three factors. We split the population in half and run a separate regression for each one. Table 3 shows summary statistics. 

Second, we consider the state of the economy. Liquidity can be effectively supplied through financial intermediaries and markets [60], risks can be diversified away, and adverse selection and morality risk, both of which are exacerbated by a lack of information, can be mitigated through financial markets and intermediaries. The E7 economic sector allows for the efficient operation of the economic intermediary and market systems. However, low-income countries struggle to build and grow high-efficiency manufacturing sectors due to the instability of their market risk diversification function [61]. In this context, the median level of financial development is used to classify the different types of financial development examined in this article. In particular, groups of nations that are above the median are considered high-level groupings, whereas groups that are [62] below the median are considered low-level. Separate regression analyses were conducted.

Finally, we consider the standard of our institutions. The research confirms the connection between financial development, economic expansion, and pollution, and the role that institutions play in each of these processes [63]. It was observed that the level of protection of investor rights differed significantly among legal systems, which in turn influenced the financial growth of nations. To better understand the impact of financial openness on economic development [64], we included financial development and social institutions in the analysis of TFP expansion. Table 4 shows cross country regression. 

We focused on China, India, Brazil, Turkey, Russia, Mexico, and Indonesia.

This research also shows that, in nations with strong institutional frameworks, financial growth may improve environmental quality by decreasing carbon emissions. Meanwhile, if a country’s institutions are weak, its economic growth may harm its ecosystems. It follows that the success of both green and financial growth depends heavily on the quality of a country’s system. Thus, this research builds a complete index of system quality using the six system quality indicators from the Global Governance Index database, referencing the work of [65]. Moreover, we categorize the sample nations into two sets, one with a higher median quality of system than the other. Those that score above the median are placed into one group, with high organizational quality, while those that score below the median are placed into another group, with poor system quality, where they undergo regression analysis in isolation. Table 5 represents the GMM analysis. 

Thus, it might be argued that the new style of urbanization has a counterintuitive impact on CO_2_ emissions, rather than a reducing one. Thus, H1 cannot be established. Three points of view are adopted in analyzing the causes [66]. First, the reductive impact of new-type urbanization has not yet materialized, since the notion of new-type urbanization is not yet thoroughly ingrained, and conventional urbanization, characterized by the construction of new cities, has not yet been altered. Furthermore, a new type of urbanization route is being established, with distinctive [67] features, and the E7 governments have not taken the necessary and optimal steps to follow this in a timely fashion. In light of the E7 governments’ recent publication of the National New-Type Urbanization and the Work Plan of National New-Type Urbanization documents, we can be confident that new-type urbanization will contribute significantly to future efforts to lower CO_2_ emissions. Lastly, the growth of new-type urbanization in E7 countries results in substantial CO_2_ emissions; however, we anticipate that the mitigating effect of new-type urbanization on CO_2_ emissions will become apparent in the not-too-distant future.

### 4.2. Robustness Analysis

At the 1% confidence level, the ES coefficients are significantly negative, indicating that the use of energy-saving technology increases [68] carbon dioxide emissions. It follows from this that the energy-saving technology does increase CO_2_ emissions, proving the existence of hydrogen gas (H_2_) [69]. This outcome may be attributed to two factors: to begin with, the increase outlined in [70]. In addition, the manufacturing factor replacement may boost CO_2_ emissions. As the DC coefficients reduce these emissions, H3 is proven [71]. We also demonstrate that the control variables affect CO_2_ emissions. There is a significantly negative correlation between tertiary industry and CO_2_ emissions, and so it may be asserted that industrial restructuring is of great importance in reducing CO_2_ emissions. However, other control variables are not statistically significant.

Although new types of urbanization have a favorable effect on carbon dioxide (CO_2_) emissions, the question of whether or not they may also influence innovation and indirectly reduce CO_2_ emissions remains unanswered [72]. It is discovered that new-type urbanization may indirectly suppress CO_2_ emissions by affecting technological growth, a finding which is demonstrated to be the case. The coefficients for variable NUES in models [73], correspondingly, are all important at the 1% level. However, the indications of the variables are mitigated as a result of new-type urbanization, and a decrease in CO_2_ emissions is achieved indirectly, proving H4. Two main causes account for this outcome: first [74]. In models A3–C3, the NUEP variable is important at the 1% level, suggesting that new types of urbanization may influence environmental [75] technologies in helping to decrease CO_2_ emissions.

### 4.3. Discussion

Businesses need to align their production practices with the growing trend of a sustainable economy if they are to succeed in today’s competitive business environment. The new form of urbanization that is propelling E7 [76] financial growth also places a premium on protecting the environment and is thus crucial to efforts to lower the country’s carbon footprint.

Although we have established the existence of EKC in other locations, the findings of this study provide strong evidence that EKC also occurs in the E7 economies. Reducing carbon emissions in the area requires a significant amount of money in order to encourage individuals and businesses to use renewable energy sources. However, for these countries to obtain the carbon neutrality goal, they must prioritize the following: increasing their GDP [77], levying environment-related taxes, fostering green technology, and investing in renewable energy R&D. Our empirical results obtained using the cross-sectionally [78] augmented autoregressive distributed lag model are supported by the results of the robustness checks (CS-ARDL).

The results of the paired panel causality tests are shown in Table 6. Both unidirectional and bidirectional causation between the considered variables have been verified by [79]. The findings outlined above highlight a unidirectional “environmental policy and carbon emission [80]”, a “composite risk index and carbon emission”, and “green technology and carbon emission”. In other words, there is no detectable feedback effect between GDP and carbon emissions. The present results are consistent with those of, who also discovered a unidirectional causality connecting GDP to CO_2_ emissions. While an enhancement in financial and commercial activity is good for the economy, the emissions caused by these endeavors contribute to environmental degradation by increasing CO_2_ levels. Research into renewable energy sources has been linked to a [81] decrease in greenhouse gas emissions, but this association appears to be one-way only. The high price tag of the necessary enhancement of R&D activities likely contributes directly to carbon emissions.

According to the research, the trade and financial sectors’ development is severely affected by ineffective and excessive energy resource consumption, which leaves the economy vulnerable to unknown external shocks (such as oil price changes or requirement mismatch worries) [82]. Increasing solid and gas emissions during manufacturing is a direct life cycle impact of wasteful power use. This means that a business will see an expansion in energy and carbon production as unsustainable energy inputs are gradually replaced with more efficient technical innovations, but also that renewable energy will be improved. Carbon pollution will be reduced thanks to powertrains.

The sensitivity of economic growth and technological innovation combined is positive and statistically significant. This study verifies the existence of a negative technology impact on financial growth and demonstrates that innovation accompanies economic advancement to raise CO_2_ emissions. In other words, financial prosperity leads to technical developments, which inevitably destroy SSA’s environment. A reasonable argument for the detrimental technological impact of financial growth is presented by [83], who claim that financial deepening drives technological advancements, which may raise the demand for renewable resources such as electricity. Conversely, the reasoning of [84] may be used to justify the adverse technological consequences of financial development. They make the argument that financial deepening exerts a negative impact on the environment through technology by offering an opportunity for companies to obtain enhanced access to large capital, which is used to help fund capital investment operations, such as purchasing more mechanical equipment, increasing the working population, and developing new factories and equipment.

Furthermore, this may lead to an increase in emissions, which degrades the quality of the environment. By considering the average, maximum, and maximum amounts of technology within the equation, we may calculate the marginal impact of economic growth on environmental quality (CO_2_ emissions) (5). Table 7 gives our conclusions on the marginal impacts of capital accumulation. We find that the incremental impact of financial progress is 0.119 at the median technology level, 1.939 at the minor technology level, and 1.01 at the highest level of technological sophistication. As a result, economic growth has little to no effect on the condition of the environment.

## 5. Conclusions

This research analyzes the changing relationship between economic growth and EF in E7 countries from 1990 to 2018. It also examines the impact of intellectual capital and FD, as well as regulatory factors and FD, on the environmental impact. At the same time, we account for electricity use. The results of CS-ARDL show that economic growth degrades environmental quality by elevating the environmental burden immediately and in the long term. On the contrary, investments in human resources and institutional factors have the potential to enhance environmental protection in the near and far future significantly. In particular, economic progress and energy utilization have a favorable influence on EF. In addition to investigating the direct impacts of economic growth on environmental quality, we also examined the resulting consequences. The identified potential between financial population and global capital demonstrates, intriguingly, that economic performance improves the sustainability of building through the human resource channel.

Conversely, financial integration moderates the effect of financial growth on environmental stewardship, which is statistically significant. The causal connection discloses the regulatory factors’ impact. This means that any legislation that addresses these issues will have an evident impact on environmental conditions. Unfortunately, these factors cannot be cancelled out by any effort to lessen the environmental impact.

We suggest several political ramifications predicated on these findings. Firstly, rising nations should enhance the current financial system, since financial growth has a catalyzing impact on ecological degradation. In this context, it is essential for E7 nations to encourage the development of novel and improved financial tools that may be used to reduce harm to the environment. On the other hand, the transfer of financial funding to polluting firms should be prevented, and more environmental initiatives should be promoted. As an economy grows and diversifies, more rules and procedures will be needed to ensure that the resulting impacts of development on the environment are kept to a minimum. This, in turn, will need constant adaptation on the financial institutions’ part to meet internal and external requirements. Secondly, social capital alleviates ecological damage, and financial growth enhances the ecological environment through the conduit of intellectual resources.

Consequently, E7 nations should invest in their citizens’ health and education. Furthermore, rising nations should also enhance the carefully placed human capital and construct a plan to address continuing pollution problems. Thirdly, there is a wide gap in the types of structures that are in place between developed and E7 nations. Nevertheless, existing data imply that institutional quality has a beneficial influence on environmental quality. In order to organize and manage environmental sustainability processes, rising nations should strengthen the ability of their administrations and endeavor to construct high-quality structures. On the other hand, powerful entities can control activities connected to banking institutions, restricting the continuous progress of damaging initiatives. Only a small number of factors have been examined, and the research solely focuses on E7 nations. Furthermore, the duration of this research was restricted to 1990–2018. Future research may enhance the model by incorporating more factors.

In the initial step, the authorities must look into refocusing FD’s conduit. One viable strategy to realize this reconfiguration is to leverage the financial mobilization channel to minimize the utilization of highly polluting technology and diesel alternatives. In this way, banking institutions may be considered intermediates to exploit the credit-providing funding route in advancing a sustainable economy. Since developing such remedies might take some time locally, governments should first import greener technology via the globalization route. These alternatives will be made accessible to the firms operating, while they will be given a limited deadline to overhaul their old technology. During the purchasing of such technologies, companies might be required to incur debts from credit intermediaries. At this time, these banking firms need to apply the discriminatory interest rate that they have determined on the carbon emissions of the enterprises, i.e., a higher cost of borrowing is to be carried by businesses with more considerable carbon emissions. This will help healthier enterprises to acquire an essential comparative benefit over their highly polluting peers in the market. This financial system will eventually exert force on the polluting enterprises, and the need for fossil energy and similar highly polluting alternatives will decline. The lower demand for coal and oil alternatives and the increasing supply of sustainable power and greener technology will eventually reduce the environmental harms associated with financial growth pathways.

After this stage is functioning, the officials should launch the second wave of the national policy. During this period, the legislators require the amplification of the generation procedure of renewable power within the country. The high costs associated with creating and deploying this method require intervention from politicians. Considering that the need for renewable energies has begun to develop throughout this phase, it will allow new businesses to join this area. These companies will inevitably need funding to keep pace with the rising demand. The regulators could help to transform the interest amount acquired during the first phase in such a way as to increase the functioning of these enterprises. If we wish to make renewable energy sources more accessible and affordable, this is the optimal stage in which to achieve this. When these multiple policy stages remain operative, the E7 nations may be able to approach the achievement of SDG 7.

Although a stepwise national policy that considers the SDGs has been proposed, it is important to keep in mind that this research has only included analysis of monetary development, the green energy demand, and intellectual resources, despite considering the moderation impacts of globalization and regulatory factors. Consequently, it might be important to evaluate the thoroughness of the regulatory framework to investigate the possibilities for improvement. Nevertheless, the difficulties mentioned in the research are highly prevalent in most emerging markets, which means that this legislative framework has adequate generalizability. This legislative framework offers a standard procedure and paradigm for other rising economies worldwide. Additionally, considering the spatial dimension of GB and CO_2_ production, this study may be of benefit to other intuitions. However, the proposed approach is sufficiently adaptable to consider policy measures situationally, allowing further study in this area.

## Figures and Tables

**Table 1:** Descriptive analysis.

Parameters	Mean	SD	CV
*TCE*	6.42	0.338	4.518
*CEPC*	15.14	2.303	16.219
*CEPPS*	10.452	6.014	14.686
*LR*	4.582	3.258	5.741
*DC*	0.245	0.472	0.016
*BC*	0.395	5.044	8.629

Notes: Mean denotes average, SD denotes standard deviation, and CV represents the coefficient of variation.

**Table 2:** Chow test for stability.

DV = Environmental Degradation	[1]
Parameters	
ln(*TCE*)*_it_*	0.503 *
ln(*CEPC*)*_it_*	0.244 *
ln(*CEPPS*)*_it_*	2.355 *
ln(*LR*)*_it_*	0.515 *
ln(*DC*)*_it_*	0.224 *
ln(*BC*)*_it_*	0.4118 *
Adjusted R-square	0.719 *
Structural Change Tests	
Chow Test (F test)	F(3.194) = 3.19 [0.0036]
Wald Test (Chi-Square Test)	8.9165 [0.0079]
LM Test (Chi-Square Test)	8.2661 [0.0081]
Country fixed effect	Yes

Note: * indicates significance level at 5%.

**Table 3:** Summary statistics.

Parameter	2005–2011	2012–2018
ln(*TCE*)*_it_*	0.5498	0.9814
ln(*CEPC*)*_it_*	0.4706	0.0652
ln(*CEPPS*)*_it_*	0.1298	0.0032
ln(*LR*)*_it_*	0.8623	0.1836
ln(*DC*)*_it_*	0.00026	0.3102
ln(*BC*)*_it_*	0.2459	0.0681

**Table 4:** Cross-country regression.

	China	India	Brazil	Turkey	Russia	Mexico	Indonesia
ln(*TCE*)*_it_*	0.1267 *	0.1179 *	0.4652 *	0.1705 *	0.0026 *	0.0698	0.070 *
	(0.1112)	(0.1231)	(0.0604)	(0.002)	(0.0097)	(0.0757)	(0.0981)
ln(*CEPC*)*_it_*	0.1714 *	0.1508 *	0.0583 *	0.1302 *	0.02128	0.02134 *	0.1417 *
	(0.1128)	(0.0561)	(0.0781)	(0.0198)	(0.0485)	(0.0131)	(0.0397)
ln(*CEPPS*)*_it_*	0.3547 *	0.017 *	0.0467 *	0.2226 *	0.1672	0.27448	0.2295
	(0.0442)	(0.0048)	(0.8082)	(0.7953)	(0.1438)	(0.1534)	(0.0194)
ln(*LR*)*_it_*	0.1464 *	0.0907 *	0.0547 *	0.4759 *	0.1447 *	0.8897 *	0.0545
	(0.45457)	(0.3197)	(0.0431)	(0.1808)	(0.1801)	(0.1296)	(0.0312)
ln(*DC*)*_it_*	0.2401 *	0.1383 *	0.0385 *	0.1904 *	0.1411 *	0.1183 *	0.0058 *
	(0.1359)	(0.1762)	(0.0036)	(0.5967)	(0.0007)	(0.0301)	(0.18813)
ln(*BC*)*_it_*	0.3098 *	0.52268	0.0182 *	0.0542 *	0.0014 *	0.9162 *	0.2113 *
	(0.5034)	(0.5273)	(0.0766)	(0.9344)	(0.0256)	(0.1939)	(0.3117)
Constant	0.8187 *	0.0643 *	0.1208 *	0.0407 *	0.9451 *	0.1827 *	0.7087 *
Country Fixed Effect	Yes	Yes	Yes	Yes	Yes	Yes	Yes
Adjusted R^2^	0.7694	0.5223	0.8727	0.8934	0.5735	0.1412	0.5673

* shows 100% significance.

**Table 5:** GMM results.

DV: Environmental Degradation		
Variable	GMM [1]	GMM [2]
ln(*TCE*)*_it_*	0.0099 *	0.6754 *
ln(*CEPC*)*_it_*	0.1809 *	0.4969 *
ln(*CEPPS*)*_it_*	0.6732 *	0.5445 *
ln(*LR*)*_it_*	0.9196 *	0.0894 *
ln(*DC*)*_it_*	0.0417 *	0.4031 *
ln(*BC*)*_it_*	0.0054 *	0.8777 *
Year—2005	0.7568 *	0.2545 *
Year—2006	0.3439 *	0.0488 *
Year—2007	0.1207 *	0.5042
Year—2008	0.3363 *	0.0036
Year—2009	0.1477 *	0.4131 *
Year—2010	0.0459 *	0.2316 *
Year—2011	0.1123 *	0.3484 *
Year—2012	0.0204 *	0.5101 *
Year—2013	0.2328 *	0.7832 *
Year—2014	0.46948	0.0027
Year—2015	0.5385	0.7812 *
Year—2016	0.2298 *	0.00876 *
Year—2017	0.2871 *	0.5557 *
Year—2018	0.0229 *	0.9886 *
Sargan test for over-identification	0.0595	0.00186

* shows 100% significance.

**Table 6:** Sensitivity analysis.

Dependent Variable Estimate	GMM [1]	GMM [2]
Short-run estimate (Environmental Degradation)	0.016452	0.00219
Long-run estimate (Environmental Degradation)	0.007666	0.002211

**Table 7:** Huber–White robust standard errors.

	China	India	Brazil	Turkey	Russia	Mexico	Indonesia
ln(*TCE*)*_it_*	0.0828 *	0.5195 *	0.9675	0.7684 *	0.1163 *	0.2036 *	0.2899 *
	(0.7245)	(0.0939)	(0.3902)	(0.0143)	(0.6214)	(0.0144)	(0.4484)
ln(*CEPC*)*_it_*	0.1605 *	0.1675	0.0459 *	0.0202 *	0.0189 *	0.2361 *	0.1559 *
	(0.0879)	(0.0017)	(0.8537)	(0.0354)	(0.0947)	(0.1481)	(0.0993)
ln(*CEPPS*)*_it_*	0.1012 *	0.0151 *	0.0594	0.0614 *	0.2562 *	0.0464 *	0.0599 *
	(0.0757)	(0.7305)	(0.1169)	(0.1505)	(0.0511)	(0.6614)	(0.0052)
ln(*LR*)*_it_*	0.3934 *	0.0748	0.0605 *	0.0116 *	0.0089 *	0.7494 *	0.1203 *
	(0.2272)	(0.0162)	(0.2208)	(0.9663)	(0.0026)	(0.0779)	(0.7038)
ln(*DC*)*_it_*	0.0297 *	0.9315 *	0.9905 *	0.6799 *	0.1396 *	0.8013 *	0.0309 *
	(0.2315)	(0.1309)	(0.0312)	(0.1957)	(0.0869)	(0.3161)	(0.0572)
ln(*BC*)_i*t*_	0.0922 *	0.0519 *	0.0666 *	0.7225 *	0.0644 *	0.1229 *	0.3738 *
	(0.8945)	(0.0097)	(0.3278)	(0.0485)	(0.5605)	(0.2773)	(0.1043)
Constant	0.0325 *	0.2969 *	0.7676 *	0.0738 *	0.2213 *	0.6603 *	0.7882 *
Country Fixed Effect	Yes	Yes	Yes	Yes	Yes	Yes	Yes

* shows 100% significance.

## Data Availability

The data used to support the findings of this study are available from the first author upon request.
